# Fundus Autofluorescence and Optical Coherence Tomography Characteristics in Different Stages of Central Serous Chorioretinopathy

**DOI:** 10.1155/2021/6649064

**Published:** 2021-05-31

**Authors:** Mary Ho, Stephanie H. W. Kwok, Andrew C. Y. Mak, Frank H. P. Lai, Danny S. C. Ng, Li Jia Chen, Lawrence P. Iu, Alvin L. Young, Marten Brelen

**Affiliations:** ^1^Department of Ophthalmology & Visual Sciences, The Chinese University of Hong Kong, Prince of Wales Hospital, Shatin, Hong Kong; ^3^Department of Ophthalmology and Visual Sciences, Hong Kong Eye Hospital, The Chinese University of Hong Kong, Kowloon, Hong Kong; ^2^Department of Ophthalmology, Caritas Medical Centre, Sham Shui Po, Hong Kong

## Abstract

**Objective:**

To describe the morphological changes on fundus autofluorescence (FAF) and spectral-domain optical coherence tomography (SD-OCT) imaging at different chronicity of central serous chorioretinopathy (CSC).

**Methods:**

This cross-sectional study included patients with CSC of different chronicity. Changes in FAF scans and morphological changes on SD-OCT were evaluated and compared at different stages of CSC.

**Results:**

Sixty-nine patients were enrolled in the study, with a mean age of 52.1 ± 11.8 years. A distinct hypoautofluorescence (AF) pattern was observed at the leakage point in acute CSC (100%). The leakage site was indistinguishable in 48% of the patients with late-chronic CSC. The majority of acute CSC patients showed hyper-AF in the area of serous retinal detachment (SRD), which persisted in the early-chronic stage of CSC. In late-chronic CSC, many cases of hypo-AF (22.2%) and mixed-pattern AF (14.8%) were observed. SD-OCT revealed evolving features of retinal pigment epithelium (RPE) abnormalities in a time-dependent manner: from peaked PEDs in acute CSC to low-lying PEDs in early-chronic CSC and, eventually, flat, irregular PEDs in late-chronic CSC. The average thickness of the photoreceptor layer (inner and outer segment; IS/OS) was 79 *μ*m in the acute group and 55.2 *μ*m in the chronic group. The photoreceptor layer (IS/OS) height was positively associated with visual acuity (*p*=0.002).

**Conclusion:**

Different stages of CSC present different patterns on FAF and SD-OCT imaging. Chronicity of CSC can be estimated using specific features in these images. Photoreceptor layer (IS/OS) height acts as a good and objective predictor of visual outcomes in CSC patients.

## 1. Introduction

Central serous chorioretinopathy (CSC) is a common condition predominantly affecting young adults. It is characterized by the collection of subretinal fluid due to choroidal vascular hyperpermeability and retinal pigment epithelium (RPE) layer defects [1]. In around 20% of the cases, the condition is recurrent or chronic in nature [2].

CSC is usually classified into acute and chronic according to the duration of symptoms [3–6]. Determining the chronicity of CSC is important in deciding the treatment plans and prognosticating visual acuity outcomes. Acute CSC usually has a self-limiting course, whereas chronic CSC with persistent serous retinal detachment has a poor prognosis. At present, there is no consensus on when treatment should be initiated in CSC patients. The first stage of management is observation for CSC resolution for a period of 3–6 months. The term “chronic CSC” generally refers to persistent CSC lasting more than 3–6 months in duration, which usually warrants clinical treatment because of the risk of irreversible visual loss. However, Singh et al. [7] reported the discordance among retinal specias in describing CSC subtypes and pointed out the need for a CSC classification system. FAF and OCT features may provide important information, supplementary to the subjective report of disease duration, which can be used to determine the chronicity of disease and provide prognostic information on the visual recovery potential.

Spectral-domain optical coherence tomography (SD-OCT) imaging can show changes in the outer retinal layers including RPE layer abnormalities, subretinal precipitates, and changes in the photoreceptor layers in chronic CSC [8–12]. Different morphologies of the pigment epithelial detachment (PED) at different stages of CSC were described by Song et al. [12]. Flat irregular PEDs in CSC were found to be associated with chronic stages of disease and a high rate of CNV detected by OCT angiography [13]. In addition, changes in the photoreceptor layer were shown to correlate with visual acuity recovery potential [14–17]. At the same time, the abnormalities of the RPE in chronic CSC can be detected by fundus autofluorescence (FAF) imaging. FAF is a noninvasive technique that determines the intensity and distribution of lipofuscin, which indirectly reflects the RPE cell function [18, 19]. Excessive accumulation of lipofuscin occurs in various retinal diseases and is believed to precede photoreceptor degeneration [20]. FAF reveals different degrees of hyper-AF in acute and early-chronic CSC cases due to the accumulated outer layer of photoreceptors not being phagocytized by macrophages [19]. In contrast, chronic cases may show different degrees of hypo-AF due to the loss of RPE cells [21, 22]. In addition, the area of hypo-AF has been reported to be related to visual prognosis of CSC patients [18, 22]. Thus, by combining both the features of OCT and FAF imaging, retinal specialists can predict the chronicity of disease and determine the necessary treatment options. This study aimed to describe the evolving OCT and FAF features from acute to chronic forms and summarize their characteristics at different stages of CSC.

## 2. Methods

### 2.1. Subjects and Methods

This cross-sectional study was approved by the ethics committee of the Hong Kong Hospital Authority New Territories East Cluster Ethics Committee governing research at the Prince of Wales Hospital, Chinese University of Hong Kong (Reference number: CRE-2015.730). All research-related investigations and procedures in this study strictly followed the guidelines of the local ethics committee. Consecutive cases of CSC at the retina clinic of our department between June 2015 to April 2017 were included. Informed consent was obtained from all patients enrolled in the study. Each patient had a documented history of CSC with symptoms of different durations and no prior treatment. The inclusion criteria were clinical diagnosis of CSC, characteristic fluorescein angiography (FA) leakage, choroidal vascular hyperpermeability on indocyanine green angiography (ICGA), and neurosensory retinal detachment with or without the presence of pigment epithelium detachment. The exclusion criteria were high myopia, glaucoma, significant media opacities, previous photodynamic therapy, coexisting retinal disease, poor-quality OCT images, and suspicion of age-related macular degeneration or polypoidal choroidal vasculopathy on FA or ICGA. Two independent retina specialists, M. B. and M. H., reviewed all FA and ICGA of included patients to rule out any case with features suggesting PCV or secondary CNV.

All recruited subjects underwent a complete ophthalmic examination including best corrected visual acuity (BCVA), dilated fundus examination, objective refraction, and axial length measurement. FAF and ICGA were performed using the Heidelberg Retina Angiograph 2 (Heidelberg Engineering, Germany) and Topcon machine (TRC-50IA Image Net, TOPCON, Tokyo, Japan), respectively.

### 2.2. Classification of Patients according to Chronicity of Central Serous Chorioretinopathy

CSC patients recruited in our study were classified according to the chronicity of disease: acute CSC (<3 months), early-chronic CSC (3–6 months), and late-chronic CSC (>6 months). Symptoms of CSC included subjective deterioration of vision, metamorphopsia, dyschromatopsia, or micropsia. Patients with recurrent disease, either by subjective symptoms or by documented medical records, were excluded from the study.

### 2.3. Fundus Autofluorescence

Autofluorescence images were acquired by retinal angiography using HRA2 (Heidelberg Engineering). Both 30-degree and 55-degree field images of the macula were obtained with an image resolution of 768 × 768 pixels. Short-wavelength FAF images were acquired by using 488 nm wavelength for excitation, and emitted light above 500 nm was detected through a barrier filter. For each image, 30 images were averaged using the inbuilt software. Hyper-AF and hypo-AF were defined as brighter and darker FAF than the intensity of the background FAF, respectively [23, 24]. The AF pattern of CSC and the intensity of AF at the site of CSC leakage were determined by two independent investigators (A.M. and S.K.). The location of leakage site and whether it was distinguishable on FAF was also assessed by the two independent investigators. The interreviewer consistency in grading the site of leakage and FAF pattern was assessed by interclass correlation.

### 2.4. Spectral-Domain Optical Coherence Tomography (SD-OCT) Images and Description of the Subretinal Pathologies

Two sets of SD-OCT images, including a volume scan and a dense raster scan of the macula, were performed. For SD-OCT images, a custom 20 × 20-degree volume-acquisition protocol of 49 sections was adopted. Dense-line scans were also performed at areas of PED. Scans with the most prominent PEDs were adopted for analysis. We classified the RPE abnormalities into four groups, taking into account reported features of different CSC stages in the literature [18–20, 25]: class 1 included peaked PED or semicircular PED; class 2 included low-lying PEDs; class 3 included flat irregular PEDs; and class 4 included RPE irregularities without evidence of RPE elevation. Particular features of the outer retina were also documented including the height of inner and outer segment (IS/OS) of photoreceptors, any sign of photoreceptor elongation or thinning, RPE layer abnormalities, and presence of subretinal hyperreflective precipitates. Morphology of the photoreceptor (IS/OS) was compared with the adjacent attached retina and adopted a similar approach to that in Piccolino et al.'s report [26]. Thickening of photoreceptor referred to increase in granulated appearance along the upper border of the subretinal fluid, while thinning referred to loss of tissue in the photoreceptor IS/OS layer.

The photoreceptor layers were described as elongated, normal, or thinned. The length of the photoreceptor inner segment/outer segment (IS/OS) was objectively measured under the fovea, with the boundaries defined as the external limiting membrane (ELM) and the retinal pigment epithelium layer, respectively; in case of the presence of subretinal fluids (SRF), the boundary was defined as the length between the ELM and upper layer of SRF. Choroidal thickness was measured subfoveally by two investigators (E.M. and F.L.) using the inbuilt caliper function of the Heidelberg Eye Explorer software. Choroidal thickness was defined as the distance between the RPE/Bruch's membrane complex and the sclera in images obtained under enhanced depth imaging mode. Mean values acquired by the two investigators were adopted for analysis.

### 2.5. Statistical Analysis

For continuous variables, the mean, standard deviation, median, range, and percentage were calculated. Analysis was performed using SPSS version 22 (SPSS Inc, Chicago, Illinois, USA). Analysis of variance (ANOVA) and Fisher's exact tests were used to compare age, visual acuity in LogMAR, and the imaging features among the three groups. Pearson's correlation analysis was performed to assess any correlation between the imaging characteristics and the clinical outcomes. Interclass correlation *κ* was adopted to assess the intergrader differences in determining the FAF pattern and leakage site. A *p* value < 0.05 was considered statistically significant.

## 3. Results

### 3.1. Demographic Data, Visual Acuity, and Ocular Features

The demographic data and clinical details of the 69 recruited subjects are summarized in Table 1. In all, 51 men (73.9%) and 18 women (26.1%) were enrolled in this study. The mean age of the enrolled patients was 52.0 ± 11.9 years (range, 21 to 82 years). The mean duration of symptoms of CSC at the time of recruitment was 4.16 ± 1.8 months. The average axial length (AL) of the affected eyes was 23.43 mm ± 0.77, and the average AL of the unaffected fellow eyes was 23.53 mm ± 1.2. There was no statistical difference in the mean AL of the affected eyes and the fellow eyes. Refractive errors of the recruited subjects showed a tendency towards hyperopia. The average spherical equivalence (SE) of the CSC affected eyes was +0.28D ± 1.32, whereas the SE of the fellow eyes was +0.49D ± 1.64.

The mean visual acuities of the acute CSC and early-chronic CSC group were 0.057 ± 0.27 logMAR and 0.111 ± 0.26 logMAR, respectively. While there was not much difference in mean visual acuity between the acute and early-chronic stages of CSC, it was significantly worse in the late-chronic CSC than in the acute stage (0.38 ± 0.39 logMAR, *p*=0.034). Correlation analysis showed a weak positive correlation between visual acuity in logMAR and duration of symptoms (*p*=0.015, Pearson's *r* = 0.377).

### 3.2. FAF Pattern at the Area of Fluid Collection

In patients with acute CSC (*n* = 8), FAF showed increased AF signal in the area of serous retinal detachment (SRD) in all eyes (100%). Of the 34 eyes with early-chronic CSC, 28 eyes showed hyper-AF (82%), and 6 eyes (17.6%) showed a mixed pattern. In the group of 27 eyes with late-chronic CSC, a substantial proportion of cases showed hypo-AF (22.2%) (Table 2).

### 3.3. FAF Pattern at Leakage Site

**Figure 1:**
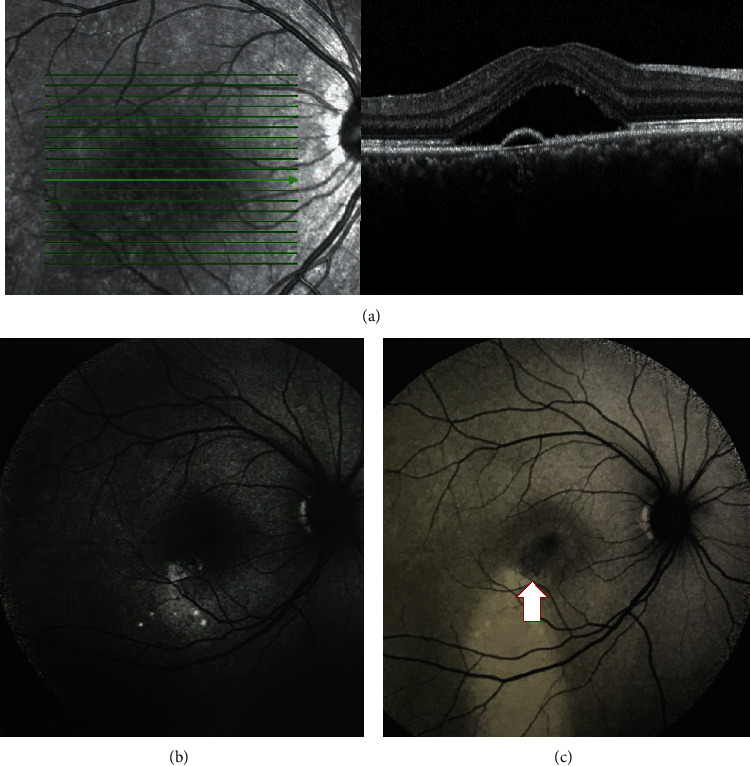
(a) SD-OCT line scan with evidence of PED at the site of leak. (b) 50-degree fundus autofluorescence scan shows a discrete hypofluorescence signal at a background of increased fluorescence at acute stage. (c) The FAF imaging of the same patient with late-chronic CSC at 6 months after symptoms onset. Given the cross-sectional study design, only the first FAF imaging and the presenting OCT scans were included for the statistical analysis. The FAF scan shows a less discrete hypofluorescence signal at the site of leak and a presence of water track sign.

In the acute CSC group (*n* = 8), FAF imaging showed a well-defined area of hypo-AF at the vascular leakage site. The leakage sites on FAF were compared to those on standard FA and ICG scans. In the acute CSC group, a small PED in combination with a discrete hypo-AF correlated well with the leakage site (Figure 1). In the early-chronic CSC group (*n* = 34), such distinct hypo-AF was less prominent than that in the acute group; 21 eyes (62%) in this group showed indistinct hypo-AF, and 13 eyes (38%) showed indistinguishable changes at the area of leakage. In the late-chronic CSC group of 27 cases, the leakage site was indiscrete and difficult to be identified on AF imaging. In 14 eyes (52%), where the leakage site could be identified, the area of hypo-FAF at the leakage site was large and not well defined. The remaining 48% of the cases showed indistinguishable patterns at the leakage site. Two independent raters were invited to evaluate the accuracy of using FAF combined with OCT images for identifying the site of leak. The consistency of different observers' assessment was assessed using intraclass correlation. The interobserver variability was *κ* = 0.714 (95% confidence interval, 0.573–0.823). Discrepancies between observers occurred at the late-chronic group, where the leakage site became less distinct or completely indistinguishable.

### 3.4. RPE Changes in Different Stages of CSC

Abnormalities of the RPE were observed in all cases. RPE abnormalities in general matched well with the leakage site in FAF or late staining site on ICG angiography. PEDs were classified into different morphology subtypes: type 1 (semicircular peaked PED), type 2 (low-lying PEDs), type 3 (flat irregular PEDs), and type 4 (RPE irregularity without definite PED).

**Figure 2:**
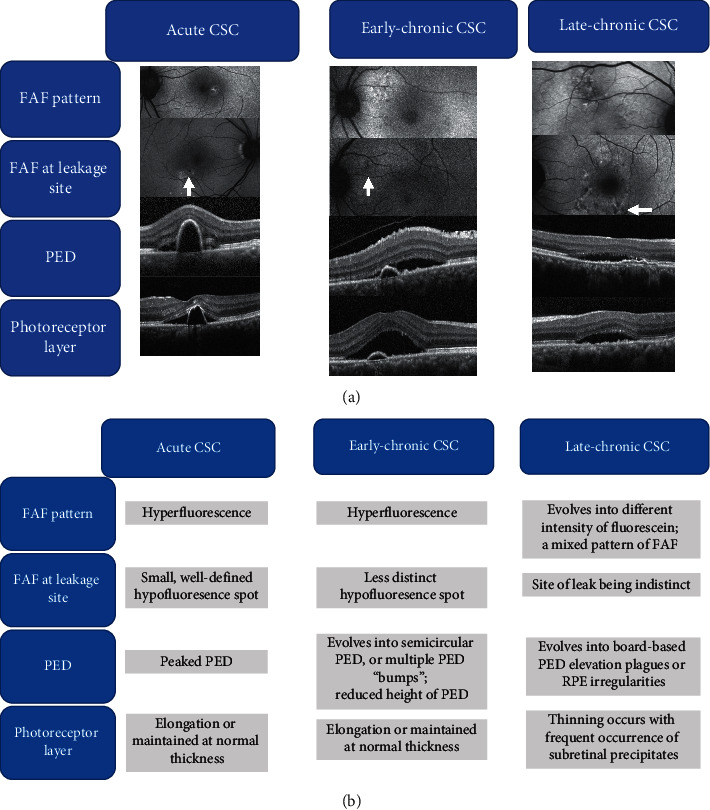
Schematic diagrams showing summary of the characteristics of (a) FAF and (b) OCT imaging changes in different stages of CSC. White arrows indicate the leakage site on the FAF images.

Peaked and semicircular peaked PEDs in the early stages of CSC correlated well with distinct hypo-FAF on the FAF scan, as well as the area of leakage on FA. Flat-irregular PEDs and raised RPE irregularities correlated well with the late staining on FA and wide area of late staining on ICG angiography in chronic stages of CSC. Peaked and semicircular peaked PEDs were commonly observed in acute CSC (33.3%); low-lying PEDs dominated early-chronic CSC (55.5%); flat irregular PEDs or raised RPE irregularities were most commonly observed in patients with late-chronic CSC (74.9%). The difference in PED patterns observed among the groups is shown in Table 2 (*p*=0.025, Fisher's exact test). The sequential change in the morphology of the PEDs is summarized in the schematic diagrams (Figures 2(a) and 2(b)).

### 3.5. Abnormalities of the Outer Retinas and the Relationship with VA

The average height of the photoreceptor layers (IS/OS) was 79 ± 13.3 *μ*m in the acute group, 72.8 ± 29.5 *μ*m in the early-chronic group, and 55.2 ± 19.9 *μ*m in the late-chronic group. There was a negative correlation between the photoreceptor (IS/OS) layer thickness and visual acuity in LogMAR (*R* = −0.464, *p*=0.002; Table 3). At the same time, a positive correlation between the photoreceptor thickness (IS/OS) and chronicity was observed (*R* = 0.377, *p*=0.001; Table 3). The reduced thickness of the photoreceptor layer (IS/OS) may serve as one of the important factors indicating visual decline. Other features noted in chronic CSC included photoreceptor outer segment thinning and subretinal precipitates. These features are summarized in Table 2. In particular, thinning of the photoreceptor layer outer segment became prominent after 3 months of CSC onset (*p*=0.002; Table 2). In addition, hyperreflective materials with subretinal precipitates and fibrinous exudates were more commonly seen in eyes with chronic stages of CSC (*p*=0.001; Table 2).

## 4. Discussion

FAF imaging provides information on lipofuscin distribution in the RPE which may occur in diseases of the outer retina and the macula [27]. The current study aimed to describe the evolving pattern of both FAF and OCT scans in CSC patients at different stages of the disease. In situations when the duration of symptoms is not available or a recall bias is suspected, these objective parameters may provide clues on the disease duration and guide treatment plans.

In the literature, much focus has been placed on evaluating FAF changes in relation to CSC's chronicity and visual outcome [22]. Zola et al. [28] reported that the pattern of granular hypo-AF is commonly observed in chronic CSC. FAF changes were also shown to be related to worse VA outcome, [18, 29, 30] as FAF indirectly reveals the functional status of the photoreceptors and RPE cells.

The FAF image reflects the health of RPE cells and, indirectly, the functional capacity of the macula. In eyes with CSC, hyperfluorescent areas are commonly seen in acute CSC, corresponding to areas of accumulated unphagocytosed photoreceptor outer segments and accumulation of lipofuscin [18, 19, 31]. This may occur because of increased shedding of photoreceptor outer segments or due to reduced function of the RPE cells. At this stage, early resorption of SRF and resolution of CSC would restore visual function. However, prominent hypo-AF may reflect areas of atrophic RPE, suggesting much guarded visual prognosis. Our findings are similar to those reported in the literature [18, 25, 31–33]. The majority of the cases in our series showed hyper-AF in the acute stage and hypo-AF or mixed-pattern AF pattern in the chronic stage. Our results, in combination with the evidence in the current literature, showed that FAF pattern evolves over time [22]. From acute to early-chronic and late-chronic CSC stages, the FAF pattern changes from hyper-AF to mixed-pattern AF to eventually hypo-AF. As hypo-AF signifies the decline in the functional capacity of the macula, a change in the FAF pattern, from hyper-AF to mixed-pattern AF, may be a good time point to consider laser treatments irrespective of the CSC duration. In addition, our study reported the OCT structural changes at different stages of CSC. In concordance with the results of Lee et al. [21], such information could provide hints on the functional status of the macula, the prognosis, and disease status and hence guide treatment planning.

In general, there is no consensus on when treatment should be initiated in patients with CSC. Some authors have defined chronic CSC as persistent fluid for more than 6 months, [6] whereas therapeutic clinical trials have used 3 months as their cut-off point [3–5, 34]. In general, the two forms of CSC behave very differently, and clinicians often rely on the history to determine when interventions need to be implemented. However, there is no definite cut-off time point to consider the initiation of treatment. After 6 months of observation, certain proportion of patients develop signs of permanent visual loss such as RPE degeneration. Clinicians could consider early intervention if signs of irreversible structural changes are noted on the FAF and SD-OCT imaging, including speckled hypo-AF and thinning of the photoreceptor layer (IS/OS).

The FAF pattern at the leakage site of CSC has been reported previously [35]. In acute CSC, focal areas of hypo-AF correspond to the site of focal RPE leakage [31]. Similar appearances of well-defined hypo-AF at the leakage site were reported by Lacono et al. [36]. In some cases, such hypo-AF may be due to focal defect of the RPE found within the PED and correspond precisely to the leakage point on FA [8, 31]. Not all eyes with CSC show hypo-AF at the leakage point [25]. We observed that such a distinct pattern of hypo-AF is obvious in early stages of CSC only. When CSC gradually evolves to the early-chronic stage, such hypo-AF changes tend to become less prominent than before. This observation was supported in our study with a high degree of interobserver agreement. We propose that, at acute CSCs, FAF, combined with OCT scans, instead of ICGA, can be employed as the guide for photodynamic laser or micropulse laser therapy. However, ICGA is valuable in certain circumstances as ICGA can reveal the choroidal vascular hyperpermeability (CVH) area, which serves as a good guidance for PDT to cover the CVH area. Also, ICGA remains the gold standard investigation when the diagnosis of CSC is uncertain, especially in suspected case of polypoidal choroidal vasculopathy or choroidal neovascularization.

The authors observed an evolving pattern of structural changes on SD-OCT evaluation in different stages of CSC. These evolving patterns of structural changes are presented in Figures 2(a) and 2(b). In eyes with acute CSC, a peaked or semicircular peaked PED is often observed at the area of leakage on FA and ICGA. These RPE elevations evolve into flat, irregular ones or mildly raised RPE plaques when the disease enters early-chronic and late-chronic stages.

This sequential RPE change could provide insight into the underlying disease mechanism of CSC. The primary pathological mechanism of acute CSC is thought to start with choroidal vascular disruption. Thereafter, the RPE decompensates allowing exudation from the choroidal vasculature into the subretinal space [37–39]. Different abnormalities in the RPE layer have been reported on OCT evaluation of CSC cases [9, 10, 40–42]. In particular, RPE abnormalities such as PED at the leakage point or within the areas of choroidal vascular hyperpermeability have been reported [8, 9]. These RPE abnormalities represent the area of RPE weakness, and the resultant RPE leak may give rise to SRD. Early in CSC development, the hypertensive choroidal perfusion pressure exerted by the pachychoroid entity leads to focal areas of RPE weakness and leakage. This effect also gives rise to the peaked PEDs and relatively large SRD in acute CSC [12]. Over time, the focal perfusion pressure appears to even out, resulting in flattened PEDs. In chronic cases, these PEDs regress to form RPE plagues or irregular RPE undulations. At the same time, with reduced abnormal choroidal pressure, the SRD generally reduces in height compared to acute stage (Figures 2(a) and 2(b)).

SD-OCT provides additional information on the microstructural morphology of the outer retina within the SRD. Several microstructural changes have been previously reported. Ojima et al., in their study, showed a persistent external limiting membrane and a disrupted IS/OS junction in the detached retina [30]. Increased thickness of the photoreceptor outer segment was reported in acute CSC [8]. In contrast, punctate or granular areas in the photoreceptor outer segment were reported in cases of chronic or recurrent CSC [26, 30]. Yu et al. [43] reported the OCT images of the outer border of the photoreceptor in CSC showing the presence of smooth, granulated, and scattered dots, reflecting different symptom durations. Similarly, Han et al. reported outer segment elongation and subretinal deposits overtime. In our study, similar microstructural changes were observed. The thickness of the photoreceptor layer (IS/OS) remained normal during the acute and early-chronic stages of CSC, but the thickness was significantly reduced in the late-chronic stage. We observed a strong negative correlation between the thickness of the photoreceptor layer (IS/OS) and duration of symptoms. In addition, a large number of subretinal precipitates were seen in late-chronic CSC. These precipitates could be attributed to the accumulation of shed or phagocytosed outer segments.

Our study has some drawbacks due to the subjective nature of FAF analysis; in addition, the duration of symptoms reported was dependent on the subjective recall of the patients. Also, the duration of the symptoms reported may not be accurate if the CSC lesion is extrafoveal. The FAF images were subjectively analyzed and an image processing algorithm was not used to determine the degree of hyper-AF or hypo-AF. It is difficult to standardize the automated averaging of images using the inbuilt FAF software. Preferably, a calibrated scale for the intensity of hyper-AF or hypo-AF at the lesion site compared to the background intensity should be used for a meaningful analysis. Secondly, the quality of FAF images is dependent on ocular media opacities and patient cooperation. Nevertheless, only high-quality images were used in this study. The measurement of the photoreceptor layer (IS/OS) was also limited. In cases of retinal layer detachment, the length of the photoreceptor layer (IS/OS) was defined as distance between the ELM and the upper layer of the SRF. When the ELM was disrupted, the measurement accuracy of the outer segment was affected. Because of the cross-sectional design, our result did not provide longitudinal data or serial scans of the same patient. Additional studies are therefore needed to investigate the fluorescence and morphological changes in FAF and SD-OCT imaging over time.

In conclusion, SD-OCT showed an evolving morphologic pattern at different stages of CSC. FAF imaging pattern in CSC eyes differed according to the time course of the disease, determined by the RPE function and outer retinal changes. Multimodal imaging including FAF and SD-OCT may provide supplementary information on the chronicity of CSC, which could help clinicians formulate management plans and prognosticate treatment outcomes.

## Figures and Tables

**Table 1:** Baseline demographic characteristics.

Mean age (years, ± SD, range)	52 ± 11.9 (21–82)
Gender	51 : 18 (M : F)
Baseline best-corrected visual acuity (LogMAR ± SD)	0.213 ± 0.303
Duration of symptom onset before presentation (months, ± SD)	4.16 ± 1.8
Average size of CSC on presentation (disc diameter, ± SD)	1.6 ± 0.84
Average mean foveal thickness CMT (*μ*m, ± SD)	389 ± 107
Refractive error (spherical equivalence ± SD)	+0.28 ± 1.32
Axial length (mm ± SD)	23.52 ± 1.2

CSC: central serous chorioretinopathy; CMT: central macular thickness; SD: standard deviation.

**Table 2:** Comparisons of the FAF and OCT findings between early-acute, subacute, and early-chronic CSC patients.

	Acute CSC (*n* = 8)	Early-chronic CSC (*n* = 34)	Late-chronic CSC (*n* = 27)	*p* value
Age (years)	40 ± 14.6	52.6 ± 8.6	54.2 ± 10.9	0.028^*∗*^^*b*^ 0.05^*∗*^^*c*3^

Visual acuity (LogMAR)	0.057 ± 0.27	0.111 ± 0.26	0.38 ± 0.39	0.034^*∗*^^*b*^ 0.033*∗*^*c*3^

Choroidal layer thickness (*μ*m)	364 ± 138.9	319 ± 47.7	337 ± 54.6	0.548 ^*b*^

*FAF findings at leakage site*				
Discrete hypo-FAF spot	8 (100%)	21 (61.8%)	14 (51.9%)	0.044^*∗*^^*a*^
Indistinct pattern at leakage site	0	13 (38.2%)	13 (48.1%)	

*Background FAF findings*				
Hyper-FAF	8 (100%)	28 (82.4%)	17 (63%)	0.020^*∗*^^*a*^
Hypo-FAF	0	0	6 (22.2%)	
Mixed-pattern AF	0	6 (17.7%)	4 14.8%)	

*OCT findings-RPE abnormalities*				
Type 1: semicircular peaked PED	4 (50%)	11 (32.4%)	2 (7.4%)	0.025^*∗*^^*a*^
Type 2: low-lying PEDs	4 (50%)	8 (23.5%)	5 (18.5%)	
Type 3: flat irregular PEDs		11 (32.3%)	9 (33.3%)	
Type 4: RPE irregularity without definite PED		4 (11.8%)	11 (40.7%)	

Height of PEDs (*μ*m)	133.3 ± 57.4	70.0 ± 5.62	72.1 ± 17.5	0.329 ^*c*1^ 0.028^*∗*^^*c*2^
Width of PEDs (*μ*m)	367.5 ± 139.2	499.3.0 ± 182.6	1297.9 ± 728.8	0.004^*∗*^^*b*^

*Abnormalities of outer retina*				
Length of the photoreceptor (IS/OS) layer (*μ*m)	79.0 ± 13.3	72.8 ± 29.5	55.2 ± 19.9	0.017^*∗*^^*b*^

*Presence of elongation or thinning of OS photoreceptor*				
Elongation	1	2	1	0.002^*∗*^^*a*^
Normal thickness	5	6	6	
Thinning	0	1	14	

Presence of subretinal precipitates	1	1	11	0.001^*∗*^^*a*^

Presence of fibrinous exudates	6	9	22	0.001^*∗*^^d^

^*a*^Fisher's exact test. ^*b*^ANOVA test (analysis of variance test). ^*c1*^*t*-test comparing acute and early-chronic groups. ^*c2*^*t*-test comparing early-chronic and late-chronic groups. ^*c3*^*t*-test comparing acute and late-chronic groups. ^*d*^Chi-Square test. *∗*, *p* value less than 0.005, with statistically significant result observed.

**Table 3:** Correlation analysis of photoreceptor (IS/OS) length, with the duration of symptoms and the visual acuity.

	Coefficient *R*	*p* value
Duration of symptoms	−0.492	0.001^*∗*^
Visual acuity in LogMAR	−0.464	0.002^*∗*^
VA versus duration of symptoms	0.377	0.015^*∗*^

*∗*, *p* value less than 0.005, with statistically significant result observed.

## Data Availability

The data used to support the findings of this study are available from the corresponding author upon request. The authors shall check with the local ethics governing committee (Hong Kong Hospital Authority New Territories East Cluster ethics committee) before releasing the data.
